# Longitudinal Variations in Antibody Responses against SARS-CoV-2 Spike Epitopes upon Serial Vaccinations

**DOI:** 10.3390/ijms24087292

**Published:** 2023-04-14

**Authors:** Dicle Yalcin, Sydney J. Bennett, Jared Sheehan, Amber J. Trauth, For Yue Tso, John T. West, Michael E. Hagensee, Alistair J. Ramsay, Charles Wood

**Affiliations:** 1 Department of Interdisciplinary Oncology, Stanley S. Scott Cancer Center, Louisiana State University Health Sciences Center, New Orleans, LA 70112, USA; dyalci@lsuhsc.edu (D.Y.); sydney.townsend14@huskers.unl.edu (S.J.B.); ftso@lsuhsc.edu (F.Y.T.);; 2 School of Biological Sciences, University of Nebraska-Lincoln, Lincoln, NE 68516, USA; 3 Department of Microbiology, Immunology and Parasitology, School of Medicine, Louisiana State University Health Sciences Center, New Orleans, LA 70112, USA; jsheeh@lsuhsc.edu (J.S.); aramsa@lsuhsc.edu (A.J.R.); 4 Departments of Medicine, Section of Infectious Diseases, School of Medicine, Louisiana State University Health Sciences Center, New Orleans, LA 70112, USA; atraut@lsuhsc.edu (A.J.T.); mhagen@lsuhsc.edu (M.E.H.)

**Keywords:** SARS-CoV-2, mRNA vaccine, VirScan, PhIP-Seq, spike, epitope map

## Abstract

The COVID-19 pandemic caused by the severe acute respiratory syndrome coronavirus 2 (SARS-CoV-2) impacted healthcare, the workforce, and worldwide socioeconomics. Multi-dose mono- or bivalent mRNA vaccine regimens have shown high efficacy in protection against SARS-CoV-2 and its emerging variants with varying degrees of efficacy. Amino acid changes, primarily in the receptor-binding domain (RBD), result in selection for viral infectivity, disease severity, and immune evasion. Therefore, many studies have centered around neutralizing antibodies that target the RBD and their generation achieved through infection or vaccination. Here, we conducted a unique longitudinal study, analyzing the effects of a three-dose mRNA vaccine regimen exclusively using the monovalent BNT162b2 (Pfizer/BioNTech) vaccine, systematically administered to nine previously uninfected (naïve) individuals. We compare changes in humoral antibody responses across the entire SARS-CoV-2 spike glycoprotein (S) using a high-throughput phage display technique (VirScan). Our data demonstrate that two doses of vaccination alone can achieve the broadest and highest magnitudes of anti-S response. Moreover, we present evidence of novel highly boosted non-RBD epitopes that strongly correlate with neutralization and recapitulate independent findings. These vaccine-boosted epitopes could facilitate multi-valent vaccine development and drug discovery.

## 1. Introduction

History is replete with examples of pandemics caused by the introduction of highly infectious agents into naïve populations [1,2,3]. Those with genetic plasticity also have the capacity to evolve and escape in the face of ever-increasing herd immunity resulting from infections or vaccinations. Coronaviruses (CoVs) have long been studied due to their high zoonotic disease and pandemic breeding capabilities. The severe acute respiratory coronavirus 2 (SARS-CoV-2) emerged as a novel betacoronavirus in Wuhan, China, in December 2019, which caused the ongoing coronavirus disease 2019 (COVID-19) pandemic and resulted in rapid spread across the world with devastating health, social and economic impacts. The pandemic produced a near all-encompassing worldwide effort to develop and implement effective vaccines, resulting in over ~13.2 billion doses administered globally as of January 2023 [4].

SARS-CoV-2 is an enveloped, positive-sense single-stranded RNA virus [5,6]. Its spike (S) glycoprotein is a major surface antigen that interacts with the host receptor, angiotensin-converting enzyme 2 (ACE2), leading to membrane fusion and viral entry [7]. The S glycoprotein is the primary target of neutralizing antibodies (nAbs) pertinent to protective immunity compared to alternative SARS-CoV-2 surface antigens, envelope (E), and membrane (M) proteins. Thus, messenger RNA (mRNA)-based vaccines encoding the S glycoprotein alone have shown efficacy in preventing the severity and fatality of COVID-19. Humoral responses to the nucleocapsid (NC) proteins can be robust following infection but are not associated with therapeutic efficacy [8,9,10,11].

Two of the most effective and frequently administered COVID-19 vaccines are the Pfizer/BioNTech BNT162b2 [12] and Moderna/NIAID mRNA-1273 [13] mRNA-based vaccines, which encode a membrane-anchored stabilized S protein through modification of two Pro mutations in the prefusion conformation [14]. Both vaccines elicit nAbs and show high efficacy in preventing severe illness, both against the original Wuhan-Hu-1 Alpha (B.1.1.7) variant (>93.8%) and the Delta (B.1.617.2) variant (88%) [15]. The Delta variant differs from the Alpha variant according to an Arg substitution for Pro at position 681 of the S protein, as a result gaining considerable replication capacity and transmissibility. To address the waning immunity against SARS-CoV-2, multi-dose vaccine regimens have been deployed. Booster doses help maintain long-term immunity and have been shown to reduce severe outcomes of the disease, as well as increase neutralization activity against variants such as Omicron [16,17,18]. In addition to the third mRNA-booster dose, which has also been shown to elicit robust Ab responses if hybrid immunity is achieved through infection prior to the booster dose [19], the current version is a bivalent booster immunization including both the initially developed S mRNA and the Omicron subvariant BA.5. The BNT162b2 vaccine has been observed to have overall lower effectiveness against the Omicron variant compared to earlier versions of SARS-CoV-2. One possible reason is a significantly higher number of Omicron S mutations, resulting in immune evasion and thereby reduced neutralizing capacity of the vaccine-induced antibodies. Multi-valent vaccine regimens thus allow for the continued protection of existing variants, as well as to acquire protection against emerging variants of concern. 

Because of the dynamic landscape of herd immunity resulting from various vaccines as well as infection coupled with the selection of escape variants with altered transmission and pathogenesis properties, it is critically important to understand how the total IgG Ab repertoires, and not just those inducing nAbs against the S glycoprotein, change with repetitive vaccinations to identify novel targets for future multi-valent vaccine development. The repertoires will also provide insight into whether de novo responses are increasing with each dose (i.e., increased breadth) or whether prior responses targeting specific epitopes are being focally reinforced (i.e., increased magnitude). Most of the cross-sectional and longitudinal studies have focused on a comparison of neutralizing antibodies or the duration of the responses across existing and novel variants of concern, yet little is known about the evolution of the responses against all potential epitopes within the S protein upon multi-dose vaccinations of the same SARS-CoV-2 strain [20]. Similarly, other studies comparing naïve or infected individuals pre- and post-vaccination have also been conducted [21,22,23,24]. However, longitudinal comparisons of the effect of multi-dose mRNA vaccinations on humoral repertoires to determine changes in the breadth and magnitude against all potential S protein epitopes are limited or absent. Information on the changes in humoral responses at an individual epitope level is important, as this will provide useful insight into the effectiveness of repeated vaccination of mRNA of the same SARS-CoV-2 spike protein since non-nAbs have also been demonstrated to have antibody-mediated effector functions that may confer protection against viral infection [25]. 

Viral epitope scanning (VirScan) utilizing the phage immunoprecipitation and sequencing (PhIP-seq) is a powerful technology that combines bacteriophage display with next-generation sequencing (NGS), enabling high-resolution profiling of antibody repertoire against all known viruses with human tropism [26,27]. Peptide and phage display libraries have already been used to characterize SARS-CoV-2 antibodies, neutralization activity, epitope patterns, and cross-reactivity in cross-sectional cohorts with mild and severe COVID-19 vs. pre-pandemic controls [28,29]. Here, VirScan was leveraged to longitudinally evaluate the breadth and magnitude of anti-SARS-CoV-2 spike (S) glycoprotein antibody (Ab) responses against all potential S protein epitopes prior to and following Pfizer/BioNTech BNT162b2 mRNA-vaccination. In addition, the Ab repertoire were compared between baseline and three post-immunization points in nine individuals without prior SARS-CoV-2 infection. Using a VirScan library presenting the tiled proteomes from 10 coronaviruses, including SARS-CoV-1 and 2, common seasonal coronaviruses (HCoV), bat CoVs (BtCoV), and MERS-CoV, we provide a high-resolution epitope map of anti-S responses and elucidate the effect of a multi-dose vaccine regimen on the breadth and magnitude of vaccine-induced responses. Furthermore, we highlight and further analyze antigenic epitopes with boosted responses and investigate neutralization data as a correlate of protection.

## 2. Results

**Figure 1:**
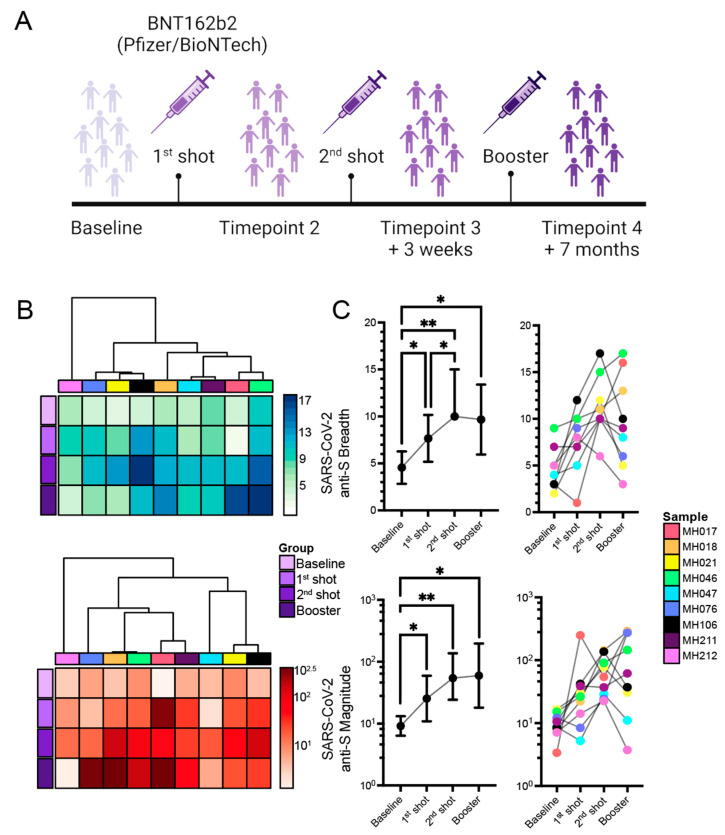
Comparing the breadth and magnitude of anti-S responses across vaccination timepoints. (**A**) Longitudinal study design. Breadth (top panel) and log-scaled average magnitude (bottom panel) of responses represented as (**B**) heatmaps with subject-level hierarchical clustering, (**C**) Median (IQR) responses (left) with before and after plots highlighting individual response patterns (right) are illustrated. 1-correlation was used as a distance measure with single linkage for hierarchical clustering of subjects. Statistically significant pairwise comparisons of vaccination timepoints are indicated with asterisks (Wilcoxon matched signed-rank test). *p*-values are denoted: ** *p* < 0.01, * *p* < 0.05.

We followed longitudinally nine SARS-CoV-2 infection-naïve individuals between the ages of 29 and 66, who are predominantly White males and females, with one Hispanic individual as an exception. All vaccinees received the monovalent BNT162b2 vaccine were enrolled before the first novel B.1.1.529 (Omicron) subvariant emerged in the US on 30 November 2021, with only one subject (MH017) as an exception, who received their booster immunization on 2 December 2021 (Table 1). Samples were collected and analyzed from baseline through three post-vaccination timepoints to interrogate epitope-level humoral responses against the SARS-CoV-2 spike (S) glycoprotein using VirScan. In addition, peripheral blood samples from the subjects were collected for immunological and virologic analyses at four timepoints: pre-vaccine baseline (timepoint 1), ~3 weeks after the first immunization (timepoint 2), ~3 weeks after the second immunization (timepoint 3), and ~3 weeks after the booster shot (timepoint 4) (Figure 1A). The Ab titers summarized in Table 1, as well as individual trends across timepoints comparing RBD and S Ab titers (Figure S1), demonstrated that vaccination produced higher titers for the whole S than the RBD region after the first and second doses of vaccination, and both showed high titers among all individuals after the booster dose. The analysis of these longitudinal samples allowed us to investigate how the breadth and magnitude of antibody (Ab) responses varied against the entire S protein prior to and after successive primary, secondary, and booster immunizations with the same antigen. 

### 2.1. Ab Responses to SARS-CoV-2 mRNA Vaccination

Antibody repertoire data was derived from subject-barcoded, high-throughput sequencing of epitope-encoding segments from immunoprecipitations of a T7 phage coronavirus proteome library using sera from vaccine recipients or no-sera controls. After selecting reactive peptides using a significance level cutoff, the data were further filtered and separated from the technical controls (mock IPs/PBS) such that there were no non-specific reactive peptides present due to spurious binding to PBS, and the anti-SARS-CoV-2 spike (S) responses of each subject and at each time point were analyzed. Out of 54 unique spike peptides contained within the VirScan CoV library, 23 (~43%) were recognized by at least one vaccinated individual; the remaining peptides were either nonreactive or did not pass the quality criteria based on PBS enrichments (see Section 4). We then calculated the breadth (i.e., the sum of reactive peptides at a given timepoint), the percent subject reactivity against each peptide, along with its average magnitude (i.e., the amplitude at which the peptide was targeted). This allowed us to compare peptide-specific immune responses across timepoints within and across subjects. 

As expected, we observed some inter-subject variation upon vaccination (Figure 1B). Specifically, there were individuals, MH018 and MH046, whose anti-S responses increased in breadth after each vaccination, indicating that they developed antibodies de novo against an increasing array of S peptides (Figure 1B). Whereas other subjects, MH017 and MH211, exhibited decreased and unchanged breadth of anti-S responses, respectively, suggesting a temporal focusing of the Ab responses against some epitopes (Figure 1B) to reflect an overall increase in magnitude. This anamnestic focus of the response was most apparent after the booster vaccination. Overall, a significant difference in the average breadth of anti-S responses across vaccination timepoints (Friedman F = 14.73, *p* < 0.01) was evident. Similar findings were observed in the magnitude of anti-S responses (Friedman F = 13.40, *p* < 0.01). Even though each dose of vaccination significantly increased the breadth and magnitude of responses in comparison to the baseline, the strongest evidence of broadening and focusing of anti-S responses was evident after the second immunization (Figure 1C).

To determine whether responses correlated with the demographic attributes of the individuals, we used generalized linear models with repeated measures to test for fixed effects such as age and sex on the differences observed in the breadth and magnitude of anti-S responses across paired samples. Fixed effects were estimated using restricted maximum likelihood estimation (RMLE). Our analyses demonstrated that there was no significant effect of age (F_breadth_ = 0.086, *p* = 0.782 [ns], F_magnitude_ < 0.0001, *p* = 0.993 [ns]) or sex (F_breadth_ = 1.866, *p* = 0.23 [ns], F_magnitude_ = 0.201, *p* = 0.661 [ns]) on the breadth and magnitude of Ab responses so that covariate adjustments for statistical comparisons across vaccination timepoints were not deemed necessary. 

#### Association of Differences of Vaccination Time or Sample Collection with Changes in Responses against the S Protein

All vaccine recipients received their first dose of vaccination within the same week, followed by their second dose three weeks post-primary immunization, booster doses were administered approximately 7 months after secondary immunization. Therefore, certain subjects with variations in the timepoints of the secondary and booster immunizations could have exerted an effect on the humoral immune response. To assess the extent to which the differences in the breadth of anti-S responses are associated with differences in the time of vaccination, we derived Kendall’s tau-b rank correlation coefficients. We did not observe any significant relationship (Kendall τ-B = −0.242, *p* = 0.1847). Similarly, differences in sample collection time did not have a significant association with differences in anti-S responses (Kendall τ-B = −0.308, *p* = 0.089). Anti-S breadth and magnitude did not display a significant vaccination time-related trend (Figure S2). Consistent observations were made when accounting for sample collection time differences. Overall, the date of vaccination or the date of sample collection had no significant association with S reactivity. 

### 2.2. Cross-Reactivities of the Anti-SARS-CoV-2 Ab Responses against Other CoVs

The VirScan library included peptides derived from the S glycoproteins of nine other coronaviruses (CoVs); therefore, we were interested in defining the extent to which vaccination with SARS-CoV-2 S mRNA induced Ab cross-reactivity against non-SARS-CoV-2 S glycoproteins. Broadly, cross-reactive antibodies could potentially be cross-protective against novel or emerging CoVs. Overall, out of the 442 unique S peptides derived from the 10 CoVs represented in the library, 206 were recognized by at least one individual. In addition, as stated in the previous section, 23 SARS-CoV-2-specific peptides were reactive in at least one individual. Interestingly, after accounting for the number of organisms and peptides represented in the library, the majority of the reactive peptides were derived from Bat coronaviruses, which are known to share the most sequence homology to SARS-CoV-2. Of the remaining peptides, we observed a similar distribution of anti-S responses among SARS-CoV-1, seasonal coronaviruses (HCoVs), and MERS-CoV. Lastly, we observed similar reactivities against all four HCoVs that commonly cause respiratory infections annually, of which the majority belonged to the HKU-N2 strain. This suggests that a single SARS-CoV-2 immunogen can induce or recall cross-reactive antibodies that could recognize human and non-human CoVs and possibly protect against or delay other zoonotic CoV infections.

Because the phage library continued peptides from entire CoV proteomes, to establish that our VirScan detection of the anti-S responses is vaccination-specific and not the result of infection or induction of anamnestic immune responses (e.g., to HCoVs), we evaluated anti-NC responses in all vaccinees against all CoV NC peptides in the library. We investigated those responses versus sequence similarity to SARS-CoV-2. Phylogenetic analyses using all Ab-reactive NC sequences supported the concept that the majority of the SARS-CoV-2-specific reactive NC epitopes shared homology with seasonal or other CoV sequences (Figure S3A). Ab titers to SARS-CoV-2 NC were low in most subjects, except in two cases where medium titers were detected after the second immunization (MH076 at 1:320) and booster immunization (MH047 at 1:640), which suggested potential SARS-CoV-2 infection during the course of the study. However, our epitope mapping did not reflect this, as the average magnitudes of subjects reacting against the NC peptides did not correlate with the NC Ab titers (Spearman r = −0.3172, *p* > 0.05 [ns]). On another note, VirScan did reveal a few high-magnitude anti-NC responses that were not from the vaccinees with medium NC Ab titers (MH106 and MH211, Figure S3B). Knowing that there would likely be some NC cross-reactivity but that such response should not increase upon serial S immunization, we were able to demonstrate that, as expected, the anti-NC magnitude of responses did not show any vaccine-associated boosting trend. In summary, responses to NC varied within and between subjects across timepoints. However, they had low magnitude, supporting that none of the individuals were infected with SARS-CoV-2 throughout the study (Figure S3B). 

### 2.3. Immunogenicity Map and Annotation of SARS-CoV-2 Spike (S) Glycoprotein-Reactive Peptides

**Figure 2:**
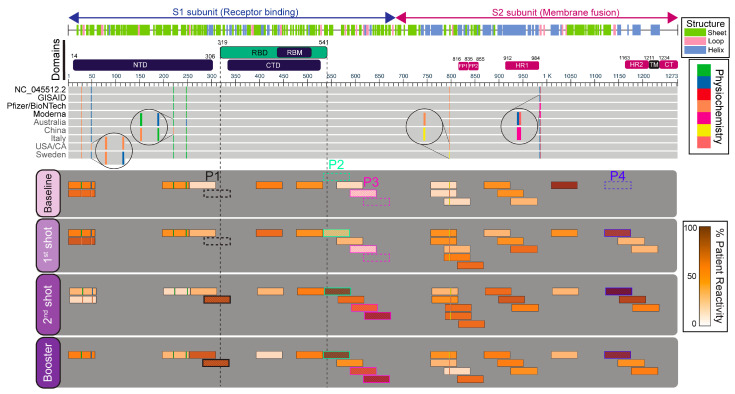
Epitope map of anti-S responses represented as percent subject reactivities. The multiple sequence alignment (MSA) of full-length SARS-CoV-2 spike glycoprotein reference, vaccine, and input library sequences are shown, where regions in light grey represent sequence conservation. Mismatched residues are highlighted and color-coded using the pre-defined Zappo scheme (green: hydrophilic, salmon: aliphatic/hydrophobic, orange: aromatic, fuchsia: conformationally special, yellow: cysteine only, red: negatively charged, blue: positively charged). Reactive peptides are mapped and colored based on % subject reactivity (brown gradient) across timepoints. Vaccine-boosted epitopes are indicated (P1–P4). Peptides with transparent backgrounds and dashed borders indicate absence of baseline responses and are only shown for convenience. Abbreviations: NTD: N-terminal domain, CTD: C-terminal domain, RBD: Receptor-binding domain, RBM: Receptor-binding motif, FP1/2: Fusion peptide 1/2, HR1/2: Heptad repeat 1/2, TM: Transmembrane, CT: Cytoplasmic tail.

Given indications that anti-S responses were the result of vaccination alone, mapping vaccine-induced immune responses to SARS-CoV-2 full-length spike protein sequences enabled us to compare the percent subject reactivities and magnitude of responses against systematically-tiled 56-mer peptides in high-resolution across multiple timepoints. We compared the distributions and changes in epitope recognition over the vaccine regimen to identify responses that boosted and their locations in the S glycoprotein. In the assessment of breadth, of the 23 reactive SARS-CoV-2 S peptides, 15 (~65%) were recognized at baseline, followed by an increase to 21/23 (~91%) after the first and booster immunizations, while 23/23 (100%) were recognized after secondary immunization (Figure 2). 

**Figure 3:**
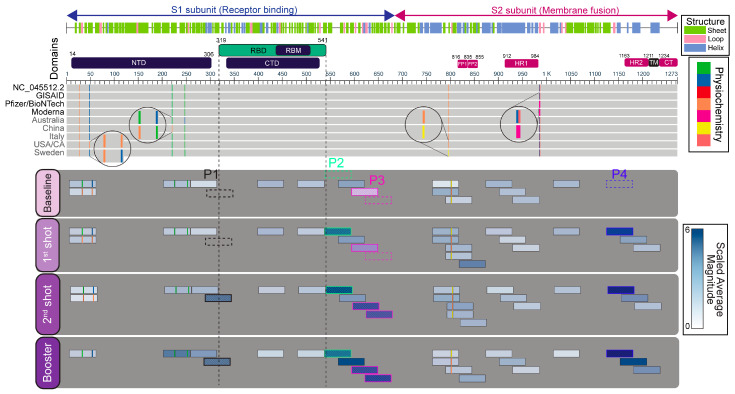
Epitope map of anti-S responses represented as scaled average magnitudes. The multiple sequence alignment (MSA) of full-length SARS-CoV-2 spike glycoprotein reference, vaccine, and input library sequences are shown, where regions in light grey represent sequence conservation. Mismatched residues are highlighted and color-coded using the pre-defined Zappo scheme (green: hydrophilic, salmon: aliphatic/hydrophobic, orange: aromatic, fuchsia: conformationally special, yellow: cysteine only, red: negatively charged, blue: positively charged). Reactive peptides are mapped and colored based on scaled average magnitudes (blue gradient) across timepoints. Vaccine-boosted epitopes are indicated (P1–P4). Peptides with transparent backgrounds and dashed borders indicate absence of baseline responses and are only shown for convenience. Abbreviations: NTD: N-terminal domain, CTD: C-terminal domain, RBD: Receptor-binding domain, RBM: Receptor-binding motif, FP1/2: Fusion peptide 1/2, HR1/2: Heptad repeat 1/2, TM: Transmembrane, CT: Cytoplasmic tail.

In order to understand why 15 anti-S responses were detected at baseline since our study subjects only consisted of infection-naïve individuals, we further investigated pre-vaccination baseline anti-SARS-CoV-2 responses for potential cross-reactivity to epitopes derived from seasonal coronaviruses. An example is a peptide, SARS-CoV-2 S aa 1008–1064, detected with sera from 8/9 individuals at baseline. However, immunization has failed to boost this response. There was a steep decline in % subject reactivity in post-immunization timepoints, and the average magnitude was consistently low at each timepoint, suggesting cross-reactivity with a prior infecting species (Figure 3). Interestingly, this peptide shared 100% sequence identity with BtCoV-RaTG13 and SARS-CoV-1 spike sequences but, on average, shared 47% sequence identity with three HCoVs. Nevertheless, the amino acids common to all recognized species are sufficient to contain at least one cross-reactive epitope. Since prior infection history with other seasonal coronaviruses was unknown, the epitope recognition diversity implicit in our limited sample size did not allow further analyses of cross-reactivity to the remaining epitopes. However, reactivities against non-boosting, pre-vaccine anti-SARS-CoV-2 epitopes indicated other peptides in the VirScan library derived from HCoV strains NL63, 229E, OC43, and HKU1-N2 were also potentially cross-reactive. Overall, no consistent pattern of boosting was observed in potentially cross-reactive epitopes.

Five reactive peptides (two overlapping) were consistently boosted among vaccinees after each dose regarding % reactivity and average magnitude. These vaccine-induced antigens are referred to as P1, P2, P3, and P4 on our epitope maps and as follows (Figure 2 and Figure 3). P1 is located between the N-terminal domain and the receptor-binding domain (RBD), with sequences overlapping both [aa 280–336]. P2 overlaps the RBD C-terminus and P3 [aa 588–672] in the S1 subunit. P3 contains two overlapping peptides, P3-I [aa 588–644] and P3-II [aa 616–672], both of which showed vaccine-boosted responses (Figure 2, Figure 3 and Figure S3). Lastly, P4 [aa 1120–1176] overlaps the heptad repeat-2 (HR2) region located within the S2 subunit, immediately N-terminal to the transmembrane domain (TM). The overlapping peptide immediately following P4 showed inconsistent % subject reactivity with lower, yet still a stepwise increase in magnitude, suggesting a de novo Ab recognition occurring at the overlapping region, whereas the N-terminus of P4 might have been an anamnestic recall response due to prior HCoV infections since it boosted with high magnitude upon the first dose of vaccination. 

**Figure 4:**
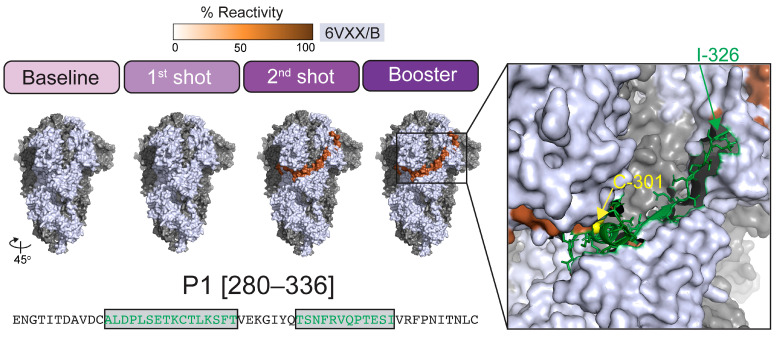
Novel boosted epitope projected on the 3D structure of SARS-CoV-2 spike (S) glycoprotein. The closed-state trimeric structure of the SARS-CoV-2 S glycoprotein was pulled from the Protein Data Bank (PDB ID: 6VXX). For brevity, only the B monomer is highlighted (light blue), and the vaccine-boosted P1 sequence was mapped onto the structure and colored based on % subject reactivity. The region with high surface accessibilities was highlighted in green and indicated on the primary sequence (boxed) and the tertiary structure.

We further explored the specificity of anti-S Ab recognition by mapping the five vaccine-induced reactive peptides’ primary sequences onto the B monomer of the 3D trimeric structure of the S protein. Consistent with Ab recognition in phage display, P1-P4 resided on the surface of the S protein (Figure 4 and Figure S3). The P1 peptide had not previously been reported as a vaccine-elicited Ab response. The P1 sequence composition was mostly hydrophilic and neutral residues (~63%). As illustrated in Figure 4, modeling of surface residues within P1 where Ab recognition would most likely occur implicated two epitopes: ALDPLSETKCTLKSFT and TSNFRVQPTESI, respectively. Interestingly, using the whole P1 sequence as input, B-cell epitope prediction using a BLAST-based module (BepiBlast) tool mapped out four potential epitopes, only one of which was highlighted as a neutralizing epitope (LKSFTVEKGIYQTSNFRV) (Figure 4). BLAST results indicated that a known epitope KSFEID**KGIYQTSNFRV**V with 82.5% sequence identity to the predicted neutralizing epitope, has been shown to mediate neutralization in SARS-CoV-1 (IEDB: 33305), where residues shared with SARS-CoV-2 are indicated in bold [30]. In P4, the linear epitope FKEELDKYFKN has been shown to be immunodominant [24,31,32,33] and to mediate SARS-CoV-1 neutralization in vitro, and is computationally predicted to do the same for SARS-CoV-2 (Figure S4) [34,35,36,37]; however, the crystal structure of this particular linear epitope was not resolved. 

Recognition of the peptides designated P2 and P3-II substantially increased among our study subjects after the second vaccination and was maintained after the booster dose. Interestingly, the overlapping peptide preceding P3-II, i.e., P3-I, elicited a response in fewer individuals compared to the P3-II. Yet, those responses still consistently boosted in magnitude across vaccination timepoints (Figure 3), suggesting that in addition to having shared sequences, the unique residues of P3-II might have elicited a separate de novo response (Figure S4). As for the overlapping region, a known immunodominant SARS-CoV-2 neutralizing epitope, reported in other studies, is highlighted in pink in Figure S4. In addition, a linear immunodominant epitope contained within P2 was also independently correlated with SARS-CoV-2 serum-neutralizing titers in vitro, highlighted in green in Figure S4 [24,29,31,32,38,39].

### 2.4. Associations of Vaccine-Induced and Non-Boosted Responses with Titer, Neutralization, and the Breadth and Magnitude of Anti-S Responses

**Figure 5:**
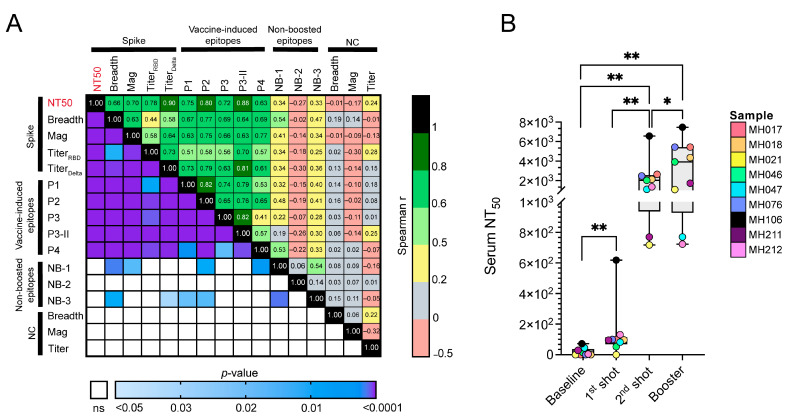
(**A**) Association of vaccine-induced (anti-S) and non-targeted (anti-NC) responses with breadth, magnitude, neutralization, and titer. The upper triangle represents the Spearman correlation coefficients, while the lower triangle represents the statistical significance of each correlation, in the order of Spike (S) and vaccine-induced S epitopes, followed by the non-boosted S epitopes, and finally non-targeted nucleocapsid (NC) breadth, magnitude and Ab titer. (**B**) Serum NT_50_ trends across timepoints. Statistically significant pairwise comparisons of vaccination timepoints are indicated with asterisks (Wilcoxon matched signed-rank test). *p*-values are denoted: ** *p* < 0.01, * *p* < 0.05.

Despite the relatively high inter-subject variation observed post-boost (timepoint 4), the breadth and magnitude of anti-S responses had a moderate-to-strong correlation. We tested the associations of the vaccine-induced responses with measured serum neutralizing titers (NT_50_) and observed strong and highly significant correlations, the highest with P3-II (Spearman CC = 0.88, *p* < 0.0001, Figure 5A). Additionally, we evaluated correlations of reactivity of three peptides that elicited non-boosting responses with low and varying average magnitudes across subjects and timepoints, with anti-NC responses as control. Two of these reactive peptides that overlap were located within the fusion peptides (NB-1: 784–840 and NB-3: 812–868), while the third was within the receptor-binding domain (RBD) [NB-2: 392–448] (Figure 2 and Figure 3). In summary, as many others have shown, boosted vaccinee responses correlated strongly with neutralization data and serum binding titer, whereas non-boosted vaccinee responses, as well as non-vaccine targets, showed weak associations (Figure 5A). Longitudinal serum NT_50_ trends show statistically significant increases in neutralization between each timepoint. However, the most drastic increase was observed after the second immunization, mirroring the breadth and magnitude of responses (Figure 5B).

## 3. Discussion

To investigate changes in the breadth and magnitude of the immune response against the S glycoprotein and whether there is any focus on the response against specific epitopes of the anti-S repertoire after three vaccine doses, a novel phage display technology (VirScan) was employed. We quantified humoral immune responses and analyzed the longitudinal reactogenicity profiles of nine SARS-CoV-2 infection-naïve BNT162b2 mRNA vaccinees. In agreement with previous findings, our pairwise comparison of the breadth and magnitude of anti-S responses showed that secondary immunization is imperative for producing optimal broadening with, on average, higher magnitudes of anti-S responses in both infection-naïve and previously infected individuals, as well as for developing stronger humoral responses against SARS-CoV-2 [40]. Additional booster immunization resulted in a consistently higher magnitude of responses compared to baseline; however, boosting was also associated with decreased overall breadth. In addition, boosting responses evinced higher variation among the subjects compared to the second immunization, supporting the concept of a personalized and diverse response compared to that following the initial two doses [41]. 

Furthermore, VirScan enabled us to generate a high-resolution epitope map of vaccine-induced anti-S responses, which then allowed us to investigate the longitudinal evolution of successive doses of vaccinations across the entire targeted S glycoprotein for all nine study subjects, both in terms of percent subject reactivities and average magnitudes that reflects the breadth and magnitude of Ab responses. We observed that the N-terminal domain within the S1 subunit (NTD, ~300 aa), which has been previously shown to have narrow specificity to neutralizing antibodies and no contribution to SARS-CoV-2 variant cross-neutralization [42,43,44], was the least recognized region of the entire S protein. Epitope maps also revealed numerous private epitopes with low specificity and magnitude spanning the fusion peptides and the heptad region 1, suggesting that the region encompassing the S2 subunit (membrane fusion) was not targeted by the subsequent vaccinations at any particular timepoint. An overlay of reactivity and magnitude maps allowed us to identify five S peptides with boosted Ab responses against the S glycoprotein. Interestingly, the consistently targeted (public) high-magnitude boosted epitopes were targets for neutralization. Of the epitopes absent at baseline that subsequently demonstrated a consistently high percent subject reactivity and a boosting response, four (P1–P4) were strongly correlated with neutralization titer (NT_50_) and have been shown by others to be associated with SARS-CoV-2 neutralization [31,33,38,39,45,46]. 

VirScan presents 56-mer peptides that are, in some cases, long enough to allow for secondary structure; nevertheless, the technique is primarily for analyses of linear or quasi-linear epitopes. Conformational epitopes may be represented, but discontinuous epitopes cannot be sampled. As a result, very little recognition against the RBD region, a subdomain known to have a complex secondary structure and shown to contain discontinuous epitopes [7], was detected. The conformational RBD epitopes are known to be more prominent targets for nAbs compared to linear RBD epitopes [32,39,47]. In this study, of the five peptides showing boosted responses, two overlapped the N- and C- termini of the receptor-binding domain (RBD), while the remaining three boosted epitopes were all located outside the RBD region, but their recognition strongly correlated with NT_50_. Consistent with the apparent importance of these non-canonical/non-RBD responses, a recent study described highly conserved coldspots across the S protein that are maintained in all SARS-CoV-2 variants that elicited neutralizing antibodies to five epitopes that overlap with the VirScan-defined vaccine-boosted epitopes (P1–P4) [48]. The exception was an epitope [aa 812–868] near the fusion peptide in the S2 subunit that did not show a stepwise increase in magnitude across post-vaccination timepoints. However, we did detect an increase in % subject reactivity post-vaccination (>50%). Our data, therefore, reinforces the importance of non-RBD anti-S humoral immune responses to vaccination, correlates of protection, and potential contributors to multi-valent mRNA vaccine design.

Because vaccinees received three identical vaccine doses (BNT162b2 mRNA), this provided an opportunity for us to comparatively evaluate associations of private/non-boosted versus boosted vaccine responses. We evaluated those associations with Ab titers, neutralization, and the breadth and magnitude of anti-S responses. At the time of construction, the VirScan CoV library did not contain the Delta or Omicron variants. Because Ab titers were obtained against the Delta variant, cross-reactivities of vaccination-induced Ab responses against Omicron and other emerging variants could not be assessed. However, our data clearly supported that the vaccine regimen produced high titer S and RBD Ab against Delta; therefore, the lack of variant sequences in the phage library did not impose drastic limitations on our findings (Table 1, Figure S1). 

Despite a small sample size admittedly not representative of the entire US vaccinated population, our overall findings highlight the identification of important longitudinally boosted vaccine-responses to SARS CoV-2 S glycoprotein. The VirScan approach revealed immunodominant linear epitopes whose recognition correlated with neutralization consistent with numerous other reports [31,33,38,39,45,46]. In addition, epitope mapping revealed private and non-nAb epitopes that demonstrated less consistency among the study subjects based on percent subject reactivity and the average magnitude of responses. Whether these epitopes play roles in antibody-mediated effector functions that provide specific individualized protection against COVID-19 requires further investigation. 

## 4. Materials and Methods

### 4.1. Subject Recruitment/COVID-19 Serological Analysis

The subject population consists of a convenience sampling of a larger study focusing on the duration of the development of serum Abs against COVID-19 proteins. All individuals underwent informed consent (approved by the Institutional Review Board of LSUHSC and the Research Review Committee of University Medical Center, New Orleans, LA, USA), 10 cc of blood was collected, and demographic information was obtained. The protocol for COVID-19 Ab testing is similar to To et al. [49]. Serum samples were tested for the presence of antibodies against SARS-CoV-2 Spike glycoprotein (receptor-binding domain, RBD) and nucleocapsid (NC) protein by an in-house assay. Optimal amounts of RBD (Arg319-Phe541) or NC protein (0.05 µg/well) of wild-type SARS-CoV-2 (Ray Biotech, Peachtree Corners, GA, USA) were placed on Immulon 2 plates (ThermoFisher, Waltham, MA, USA) in 0.9 M Sodium carbonate buffer (pH 9.5), blocked (10 mM Tris, 0.15M NaCl, 0.5% Tween-20, 10% goat serum) and sera added at 2-fold dilutions starting at 1:10, and detected with goat-anti-human IgG (H and L) antibody (Invitrogen, Boston, MA, USA). Negative controls from at least 5 individuals were derived from archived serum samples collected prior to 2015. Positive controls from at least 5 individuals were derived from a bank of discarded unidentified sera that reacted strongly in the assay. End-point dilution titers were defined as the lowest dilution of sera that produced a signal greater than 3 standard deviations over the average of the negative controls. Seropositivity was defined as an end-point dilution titer greater than 1:80.

### 4.2. Phage Immunoprecipitation and Sequencing (PhIP-Seq)

The T7-HCoV-56-mer phage library was generously provided to us by Dr. Stephen J. Elledge at Harvard Medical School, and it was previously described [29]. Phage immunoprecipitation and sequencing (PhIP-Seq) have been previously described by us and others [26,27,29,50]. Briefly, the library was amplified and titered per the manufacturer’s instructions (Novagen T7Select System, Burlington, MA, USA). Then, 1.4 × 10^9^ pfu of the library (≈2 × 10^5^ pfu/library member) was mixed with plasma containing 2 µg of total IgG. The phage–antibody complexes were immunoprecipitated using Protein A and Protein G magnetic beads (10008D/9D, Invitrogen, Boston, MA, USA) and a magnetic separation rack (S1511S, NEB, Ipswich, MA, USA). After immunoprecipitation, the beads were resuspended in 40 µL of nuclease-free water, heated to 95 °C to lyse the phage, and the phage DNA was PCR amplified. During PCR amplification, each well of the 96-well plate was barcoded, and Illumina adaptors were added. The 376bp amplicon was then gel-extracted and sent to the UNMC Genomics core for quality check and sequencing. Finally, the clustering and sequencing were performed on an Illumina NextSeq550 using the 50-cycle, single-end protocol (Mid-output flow-cell). The run was monitored by Illumina Sequence Analysis Viewer, and the final FASTQ files were generated after de-multiplexing.

### 4.3. Pseudovirus Production and Neutralization Assays

To generate SARS-CoV-2 Spike pseudotyped virus lots, Lenti-X 293T cells (Takara Bio, San Jose, CA, USA) were cultured in Dulbecco’s modified eagle medium (DMEM) supplemented with 10% fetal bovine serum (FBS), 10 mM HEPES, 1 mM sodium pyruvate, 1x MEM non-essential amino acids, and 1% penicillin/streptomycin in T75 tissue culture flasks in a humidified 37 °C, 5% CO_2_ incubator. When the monolayers reached 50–80% confluency, cells were transfected with 0.53 μg Wuhan-Hu-1 Spike-encoding plasmid (Genbank no. NC_045512, synthesized at Genscript), 9.2 μg pHR’CMV-Luc (VRC5601, NIH Vaccine Research Center), 9.2 μg pCMV ΔR8.2 (VRC5602), 0.16 μg TMPRSS2-encoding plasmid (VRC9260). These four plasmids were delivered using the jetPRIME transfection reagent (Polyplus), according to manufacturer’s protocol. At 96 h post-transfection, culture supernatants were harvested, clarified, and stored at −80 °C.

Permissive HEK-293T-ACE2 cells (BEI Resources, NR-52511) were seeded at 3.0 × 10^5^ cells/well in 96-well black wall, clear-bottom assay plates (Corning, Corning, NY, USA) six hours prior to beginning the neutralization assay. BNT162b2 vaccinee sera samples were serially diluted 1:3 in a culture medium (starting at 1:30 dilution) in sterile 96-well round-bottom tissue culture plates (Corning, Corning, NY, USA). SARS-CoV-2 Spike (Wuhan-Hu-1) pseudovirus was added 1:1 (*v*/*v*) to diluted sera samples and incubated at 37 °C for one hour. Permissive cell seeding volumes were aspirated from the assay plates, and the pseudovirus/sera mixtures were transferred to appropriate assay wells. At 24 h post-transduction, all assay wells were replenished with fresh culture medium (0.2 mL/well). At 72 h post-transduction, assay wells were developed using the ONE-Glo Ex Luciferase Assay Kit (Promega, Madison, WI, USA), according to manufacturer’s protocol. Luciferase reporter signaling events were detected using the Cytation1 multi-mode reader (BioTek, Winooski, VT, USA).

### 4.4. Data Processing and Statistical Analyses

The database that contains phage library annotations includes but is not limited to, the amino acid sequence and taxonomic identification for each isolate from strain to kingdom levels, integrated with the UniProtKB identifiers. As previously defined, each peptide is represented with a unique identifier based on their oligonucleotide sequences, such that raw counts obtained from peptides with identical amino acids can be consolidated to prevent misrepresentation of the quantified Ab responses and consequently the breadth and magnitude of responses [50]. Additionally, peptides corresponding to obsolete, redundant, or deleted UniProtKB IDs were either updated or removed from downstream analyses. Lastly, the database was further manually curated for consistent protein names within and across organisms.

PhIP-seq data was analyzed as previously defined, with slight modifications [50]. Briefly, the sequence reads were aligned, and PhIP-stat tool (https://github.com/lasersonlab/phip-stat, accessed on 28 June 2022) was used for generating the raw count data along with the Gamma-Poisson fitted residual *p*-values for each peptide. Using the −log_10_(*p*-value), or MLXP, we defined the magnitude of Ab responses, representing the frequency with which a peptide is targeted. To achieve maximal depth before any comparative analyses with a VirScan library encompassing only ten CoV organisms, we conducted duplicate IP with patient sera and merged the sequence dataset to create a single peptide reactome per subject. Based on a significance cutoff using the magnitude of responses (i.e., MLXP > 1.3, or *p* < 0.05), we identified the *reactive* peptides, indicating that the plasma had an antibody that confidently reacted against the given peptide. The sum of reactive peptides per protein or proteome per sample represents the breadth of Ab response. Additional to this statistical cutoff, we employed quality cutoff criteria for low-quality peptides, i.e., peptides that showed spurious non-specific binding to technical controls (mock IPs/PBS). We removed the peptide from our analyses if it was reactive in >2/8 (25%) of the PBS samples. All statistical analyses were performed in R v.4.2.0 and GraphPad Prism v9.3.1. 

### 4.5. Epitope and Structural Mapping

In order to map peptides for longitudinal comparison in terms of % subject reactivity and magnitude of Ab responses, full-length protein sequences of SARS-CoV-2 S glycoprotein isolates present in the CoV VirScan library were obtained from the Protein Knowledgebase (UniProtKB). Additionally, reference (NC_045512.2 and GISAID) and vaccine S sequences (both Pfizer/BioNTech BNT162b2 and Moderna/NIAID mRNA-1273 were included in the multiple sequence alignments (MSA), using the constraint-based alignment tool (COBALT), employing high gap introduction and extension penalties, and without query clusters. The trimeric structure of the SARS-CoV-2 S glycoprotein was pulled from the Protein Data Bank (PDB ID: 6VXX, closed state), and further analyses and visualizations were performed in PyMOL™ v2.5.2 using custom scripts.

## Figures and Tables

**Table 1:** Study subjects’ demographics and clinical characteristics (*n* = 9). Demographics, such as age and sex, as well as Ab titers, serum neutralization, and sequence data, are shown across each of the four timepoints. Ab titers were categorized as below limit of detection (≤ 1:40), low (>1:40, <1:160), medium (≥ 1:160, <1:1280), and high (>1:1280). Significant pairwise comparisons across vaccination points are denoted: ^A^ Baseline vs. 2nd shot, ^B^ Baseline vs. Booster, ^C^ 1st shot vs. Booster, ^D^ Baseline vs. 1st shot. *p*-values are denoted: **** *p* < 0.0001, *** *p* < 0.001, ** *p* < 0.01, * *p* < 0.05. Abbreviation: SD—standard deviation; LOD—limit of detection; IQR—Inter-quartile range, W—White; H—Hispanic.

Demographics	Baseline (*n* = 9)	1st Shot (*n* = 9)	2nd Shot (*n* = 9)	Booster (*n* = 9)
**Age**|Mean (SD)	47 (13.23)	–	–	–
**Sex**|Male (Female)	5 (4)	–	–	–
**Race|**n	W (8), H (1)	–	–	–
**Sample Collection**	May 20–January 21	February 21	February–March 21	October–December 21
**Vaccination**	–	January 21	February 21	October–December 21
**Antibody**				
**RBD/Spike Titer** ^B^****^, C^**	*n* (max %)			
Below LOD	7 (78%)	1	1	0
Low	0	1	0	0
Medium	2	6 (67%)	3	0
High	0	1	5 (56%)	9 (100%)
**NC Titer**	*n* (max %)			
Below LOD	6 (67%)	7 (78%)	5 (56%)	6 (67%)
Low	3	2	3	2
Medium	0	0	1	1
High	0	0	0	0
**Delta Titer** ^A^***, ^B^***	*n* (max %)			
Below LOD	6 (67%)	1	0	0
Low	1	1	0	0
Medium	2	4 (45%)	1	0
High	0	3	8 (89%)	9 (100%)
**Total IgG** ^A^**, ^B^*, ^D^**				
Median (mg/mL) (IQR)	3.178 (2.070)	2.024 (1.740)	2.013 (0.633)	1.949 (2.492)
**Neutralization** ^A^**, ^B^*, ^C^****				
Serum NT_50_|Mean (SD)	18.151 (25.302)	141.159 (182.859)	2199.893 (1785.858)	3433.839 (2451.889)
**Sequencing Data**				
**Total counts/Replicate**				
Median (IQR)	1,576,212 (201,681.25)	1,640,594.5 (118,177.75)	1,604,607 (179,009.5)	1,569,583 (166,601)
**Fraction of aligned reads (%)**	>97%	>97%	>97%	>97%

## Data Availability

The data presented in this study are available upon request.

## Supplementary Materials

The supporting information can be downloaded at: https://www.mdpi.com/article/10.3390/ijms24087292/s1.
