# Supplementary material for: Longitudinal Variations in Antibody Responses against SARS-CoV-2 Spike Epitopes upon Serial Vaccinations
Source: Int J Mol Sci. 2023 Apr 14;24(8):7292. doi: 10.3390/ijms24087292 (PMC10138620; doi:10.3390/ijms24087292)
Supplement: Supplementary file 1 [file ijms-24-07292-s001.zip › ijms-2286998-supplementary.pdf]

Supplementary Materials for**:**

Longitudinal variations in antibody responses against SARS-CoV-2 spike epitopes upon serial vaccinations

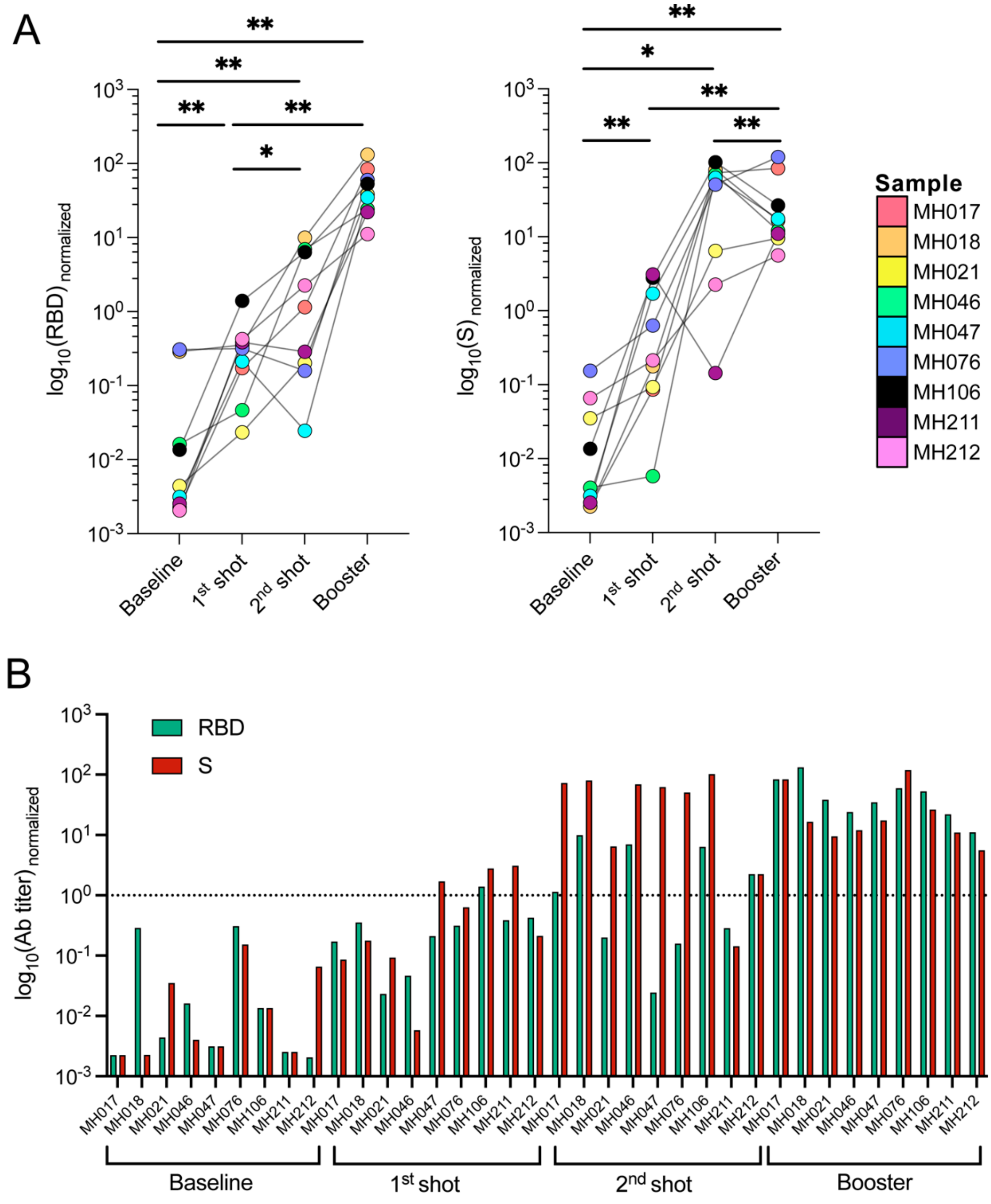

Figure S1: Total IgG normalized Ab titers obtained by binding-assays (ELISA) for RBD and S region of the Delta variant. (A) Statistical comparisons across timepoints for log-scaled normalized RBD and S regions Ab titers, with individuals highlighted. Statistically significant pairwise comparisons of vaccination timepoints are indicated with asterisks (Wilcoxon matched signed ranked test). *p*-values are denoted: $^{**}p<0.01$, $^{*}p<0.05$. (B) Comparison of RBD and S Ab titers for each individual across vaccination timepoints.

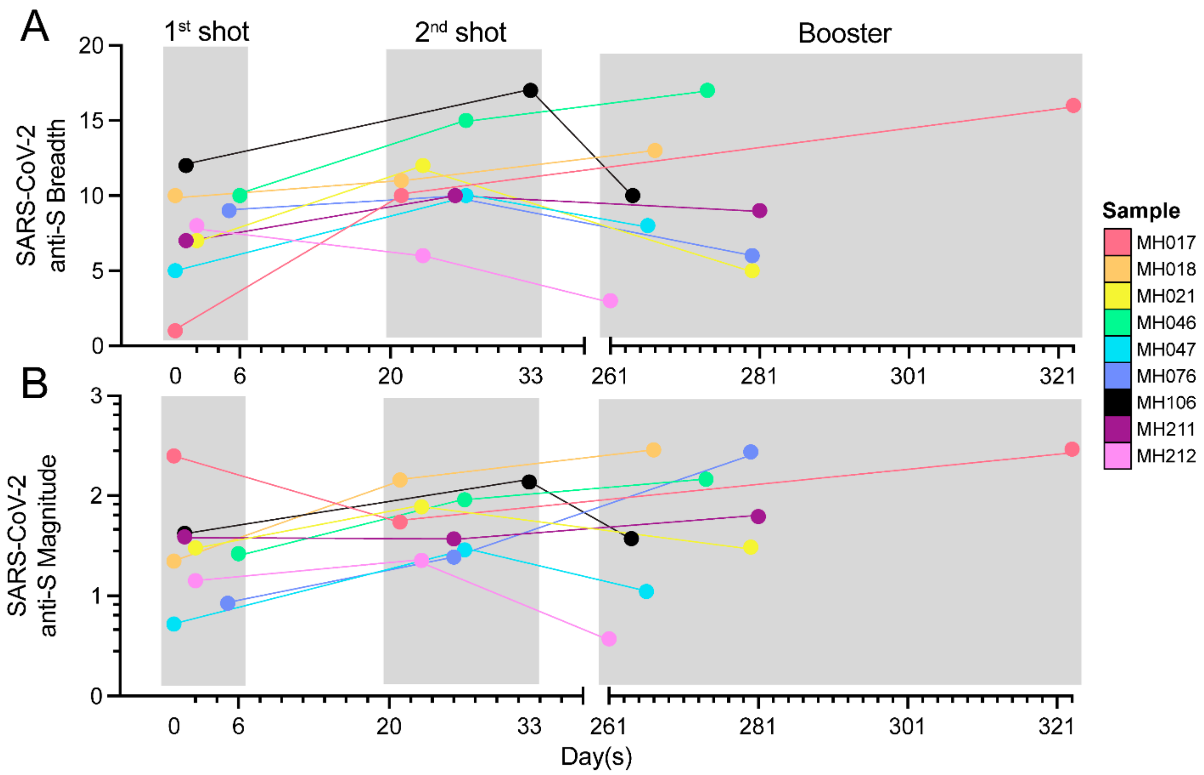

Figure S2: Patterns of anti-S breadth and magnitude of responses with respect to vaccination time across individuals. (A) Breadth and (B) magnitude of anti-S responses of each vaccinee are plotted against time, represented as days. Days are calculated per individual relative to the date of each of their initial dose of vaccination, represented as day 0.

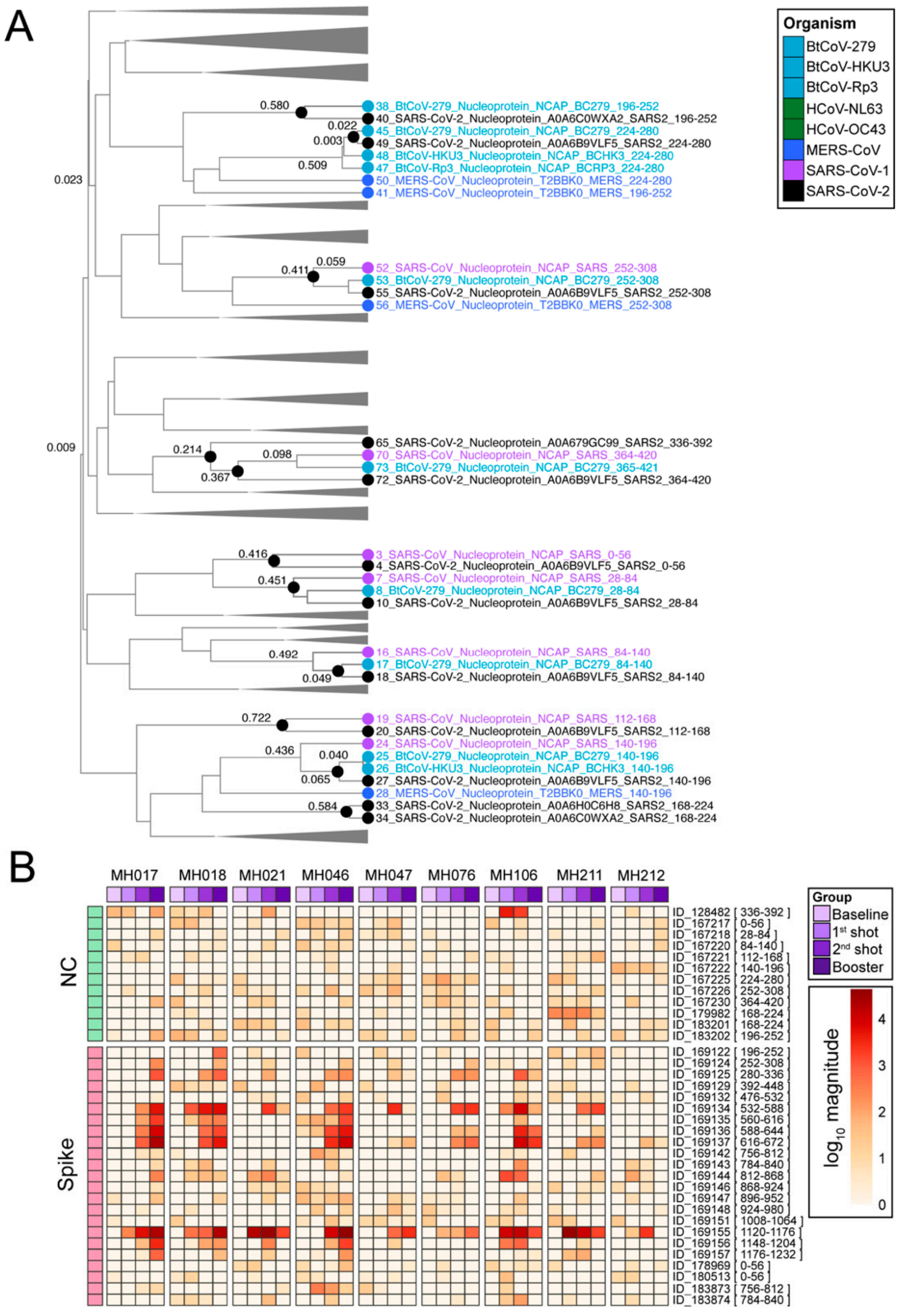

Figure S3: Investigating cross-reactivity of non-targeted responses in SARS-CoV-2 naïve individuals. (A) Hierarchical clustering of reactive nucleocapsid (NC) peptide sequences (56-mer) from all CoVs, color-coded by organism. Clades that did not include SARS-CoV-2 are pruned (represented as the grey triangles). (B) log-scaled magnitudes of NC and spike (S) reactive peptides for each individual across timepoints. Each peptide is represented by its ID, with relative start and end location within its protein.

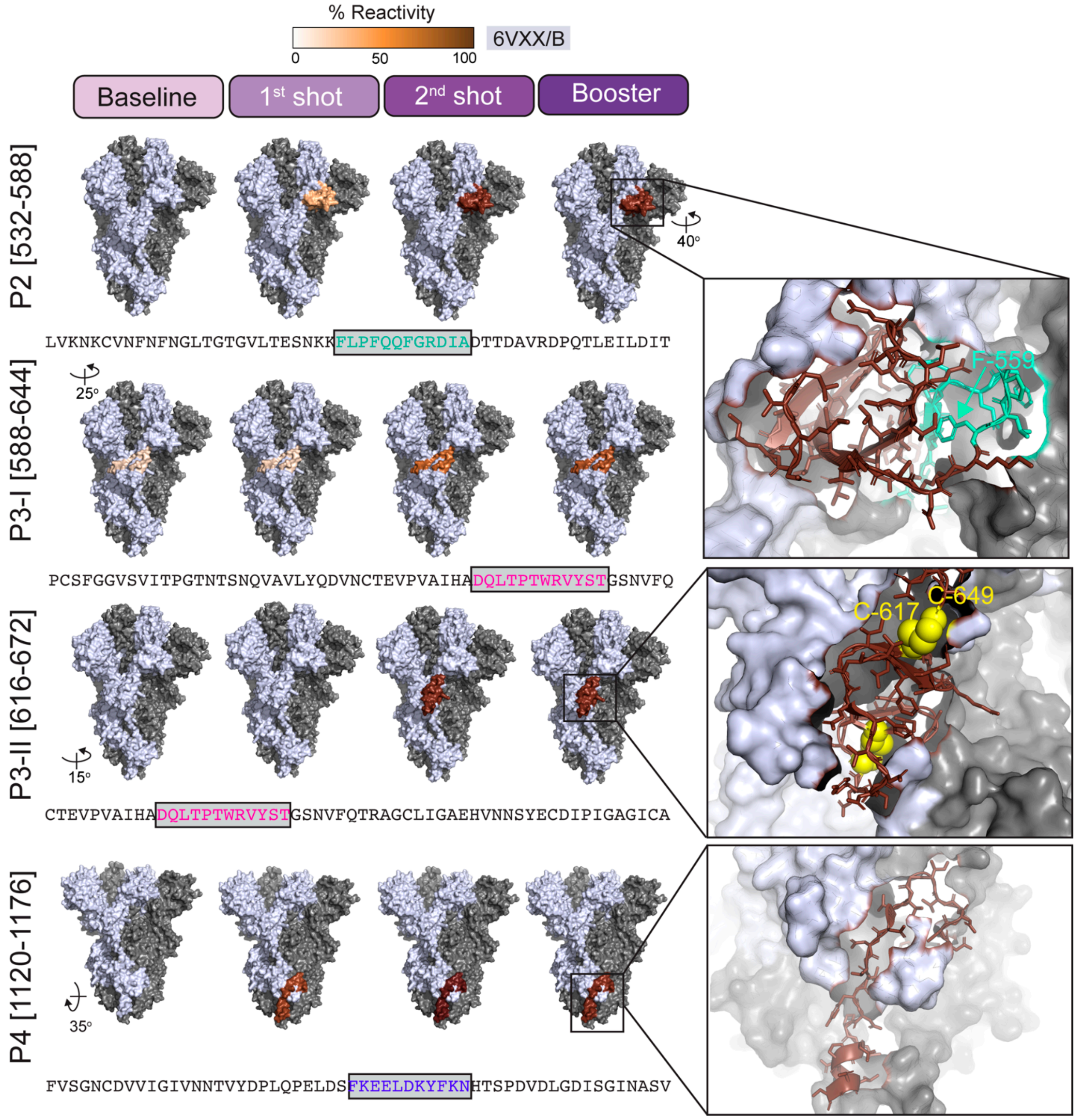

Figure S4: 3D projections of remaining vaccine-induced epitopes that are boosted across immunization timepoints that are also supported by other studies. The closed state trimeric structure of the SARS-CoV-2 S glycoprotein was pulled from the Protein Data Bank (PDB ID: 6VXX). For brevity, only the B monomer is highlighted (light blue), and the vaccine-boosted sequences were mapped onto the tertiary structure and colored based on % subject reactivity. The subsequences with known neutralization activities based on independent findings were highlighted and boxed within each primary sequence of the vaccine-boosted peptide.
